# Behavioral and inflammatory response in animals exposed to a low-pressure blast wave and supplemented with β-alanine

**DOI:** 10.1007/s00726-017-2383-8

**Published:** 2017-02-04

**Authors:** Jay R. Hoffman, Amitai Zuckerman, Omri Ram, Oren Sadot, Jeffrey R. Stout, Ishay Ostfeld, Hagit Cohen

**Affiliations:** 1 Institute of Exercise Physiology and Wellness, Sport and Exercise Science, Burnett School of Biomedical Sciences, University of Central Florida, Orlando, FL 32816 USA; 2 Division of Psychiatry, Anxiety and Stress Research Unit, Faculty of Health Sciences, Beer-Sheva Mental Health Center, Ben-Gurion University of the Negev, Beer-Sheva, Israel; 3 Department of Mechanical Engineering, Ben-Gurion University, Beer-Sheva, Israel; 4 Israel Defense Forces, Medical Corps, Tel Aviv, Israel

**Keywords:** Supplementation, Carnosine, Mild traumatic brain injury, GFAP, BDNF

## Abstract

This study investigated the benefit of β-alanine (BA) supplementation on behavioral and cognitive responses relating to mild traumatic brain injury (mTBI) and post-traumatic stress disorder (PTSD) in rats exposed to a low-pressure blast wave. Animals were fed a normal diet with or without (PL) BA supplementation (100 mg kg^−1^) for 30-day, prior to being exposed to a low-pressure blast wave. A third group of animals served as a control (CTL). These animals were fed a normal diet, but were not exposed to the blast. Validated cognitive-behavioral paradigms were used to assess both mTBI and PTSD-like behavior on days 7–14 following the blast. Brain-derived neurotrophic factor (BDNF), neuropeptide Y, glial fibrillary acidic protein (GFAP) and tau protein expressions were analyzed a day later. In addition, brain carnosine and histidine content was assessed as well. The prevalence of animals exhibiting mTBI-like behavior was significantly lower (*p* = 0.044) in BA than PL (26.5 and 46%, respectively), but no difference (*p* = 0.930) was noted in PTSD-like behavior between the groups (10.2 and 12.0%, respectively). Carnosine content in the cerebral cortex was higher (*p* = 0.048) for BA compared to PL, while a trend towards a difference was seen in the hippocampus (*p* = 0.058) and amygdala (*p* = 0.061). BDNF expression in the CA1 subregion of PL was lower than BA (*p* = 0.009) and CTL (p < 0.001), while GFAP expression in CA1 (*p* = 0.003) and CA3 (*p* = 0.040) subregions were higher in PL than other groups. Results indicated that BA supplementation for 30-day increased resiliency to mTBI in animals exposed to a low-pressure blast wave.

## Introduction

Traumatic brain injury (TBI) occurs when an external force results in temporary or permanent neurologic dysfunction. TBI resulting from explosions have been reported to be the most common injury experienced by combat soldiers injured in the recent conflicts in Iraq and Afghanistan (Warden 2006). These injuries can range in spectrum from mild to severe, often from exposure to a high pressure blast. However, exposure to a low-pressure blast wave can also exert significant forces on brain tissue without actual penetration of the skull, but still result in changes in neural function (Mayer et al. 2011). These injuries are on the mild end of the TBI spectrum (mTBI) and are the most common form (~75%) of TBI in soldiers returning from combat operations (Warden 2006; Marshall et al. 2012).

Most events leading to TBI/mTBI are also traumatic, and the symptoms associated with mTBI are at times difficult to distinguish clinically from post-traumatic stress disorder (PTSD) (Bryant et al. 2010; Elder et al. 2012; Ojo et al. 2014; Pape et al. 2013). Recently, it has been suggested that some neurocognitive effects associated with blast exposure, may be better explained by PTSD symptom severity rather than blast exposure, or mTBI history alone (Storzbach et al. 2015). In both disorders complaints of fatigue, irritability, and poor sleep are frequent. Impaired concentration, attention, and memory are also common, and neuropsychological test profiles may look similar between both disorders with deficits seen in attention, working memory, executive functioning, and episodic memory (Bryant et al. 2010; Elder et al. 2012; Ojo et al. 2014).

A recent investigation from our group demonstrated that β-alanine supplementation for 30-day in a rodent model was able to increase resiliency to PTSD (Hoffman et al. 2015b). This was thought to be related to elevations observed in brain carnosine, and subsequent protection of brain-derived neurotrophic factor (BDNF) expression in the hippocampus. These changes were consistent with others that have demonstrated an association between elevations in brain carnosine content and BDNF expression (Murakami and Furuse 2010). The mechanism of elevated brain carnosine and maintenance of BDNF expression in the hippocampus is not well-understood, but it may be related to carnosine’s role as a neural protectant through its action as an antioxidant (Kohen et al. 1988). Oxidative stress and inflammation in the brain have been suggested to be part of the sequelae of physiological events contributing to PTSD (Wilson et al. 2013), but may also contribute to the cognitive and neurodegeneration associated with mTBI (Aungst et al. 2014; Perez-Polo et al. 2015; Yang et al. 2013). Elevation in brain carnosine may increase antioxidant capacity potentially preventing neurodegeneration associated with the inflammatory response. In addition, reducing the oxidative response may also contribute to preserving BDNF expression.

Neuropeptide Y (NPY) is a neuropeptide that is widely distributed in the central nervous system, and has also been suggested to regulate the emotional response to stress (Heilig 2004), and is also associated with learning and memory (Gotzsche and Woldbye 2016). Recent investigations have demonstrated that NPY regulation is associated with behavioral resilience to stress in rodent models of PTSD (Cohen et al. 2012; Hoffman et al. 2015a). One of the primary roles of BDNF and NPY is to provide neuroprotection and/or neurotrophic action in various brain segments (Cowansage et al. 2010; Croce et al. 2012). A decrease in cognitive function and memory is a clinical feature associated with mTBI that may result from a dysregulation of neurotrophin expression (Kaplan et al. 2010). Both mTBI and PTSD are disorders characterized by overlapping neural mechanisms involving alterations in dendritic remodeling, and neurogenesis within the hippocampal and prefrontal cortical regions. Although the mechanism is not completely understood, evidence does suggest that elevations in cytokine inflammatory markers may contribute to neuronal disruption in rodent models of mTBI (Perez-Polo et al. 2015; Yang et al. 2013). During a traumatic event, microglia are activated from a resting state and migrate to the site of injury (Jacobowitz et al. 2012). The activated microglia are thought to have a phagocytic role and potentially release a variety of inflammatory mediators at the site of injury (Jacobowitz et al. 2012). Glial fibrillary acidic protein (GFAP) is an astrocyte protein that is elevated following brain injury (Hylin et al. 2013; Kochanek et al. 2006; Perez-Polo et al. 2015; Yang et al. 2013). Elevations in GFAP are thought to be representative of astrocytes providing trophic support to assist in the recovery processes (Dougherty et al. 2000). Blast associated neurodegeneration has also been associated with an increased expression of phosphorylated tau protein (Du et al. 2016; Goldstein et al. 2012). Mechanisms resulting in increases in tau protein expression are not well-understood, but are thought to be related to increases in inflammation (Bhaskar et al. 2010).

The primary purpose of this study was to examine the effect of β-alanine ingestion on behavioral and cognitive responses relating to mTBI and PTSD in rats exposed to a low-intensity blast wave. In addition, we investigated the effects of β-alanine ingestion on inflammatory, neurotrophin and tau protein expression in the hippocampus.

## Methods

### Animals

Adult male Sprague–Dawley rats weighing 200–250 g were habituated to housing conditions for at least 7 days. All animals were housed four per cage in a vivarium with stable temperature and a reversed 12-h light/dark cycle, with unlimited access to food and water. In animals randomized to the supplement group (BA), β-alanine was provided with glucomannan in a powder form (80:20 blend). Rats were provided with 100 mg of the powder per kg of body mass (a total of 30 mg of powder was dissolved in 25 ml of water). PL treated rats were provided with the vehicle (glucomannan) at the same relative dose. Animals were handled once daily. All testing was performed during the dark phase in dim red light conditions. This study was performed according to the principles and guidelines of the National Institute of Health Guide for the Care and Use of Laboratory Animals. All treatment and testing procedures were approved by the Animal Care Committee of the Ben-Gurion University of the Negev, Israel.

### Experimental design

Rats were randomly assigned to one of three treatment groups:

1. Vehicle-treated group + blast (PL; *n* = 50): rats were fed regular food and water for 30 days and exposed to the low-pressure blast wave.
2. β-Alanine + blast (BA; *n* = 49): rats were fed regular food, provided β-alanine in their water for 30 days and exposed to the low-pressure blast wave.
3. Vehicle-treated + unexposed (CTL; *n* = 10); rats were fed regular food for 30 days and were not exposed to the low-pressure blast wave. These animals served as controls for immunofluorescence analysis.

Following the 7-day acclimation period in which all rats received a normal powder diet, they were randomized into the three groups. Following 30-day of either a normal diet or a β-alanine supplemented diet the rats in BA and PL were exposed to a low-pressure blast wave. Diets were maintained until the end of the study.

**Fig. 1:**
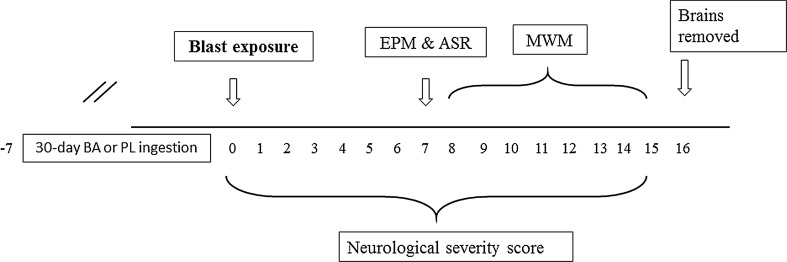
Study design. Succession of events over the time line (*arrow*). *EPM* elevated plus maze, *ASR* acoustic startle response, *MWM* Morris water maze

Neurological assessment using the neurological severity score (NSS) was performed 1 h following the blast and daily thereafter. Behavior measures were conducted on day seven following the blast. Animals were initially assessed in the elevated plus maze (EPM) followed by the acute startle response (ASR) paradigm 1 h later. Spatial memory performance using the Morris water maze (MWM) test was assessed at 8 days post-exposure, for eight consecutive days. Rats were killed 24 h following the completion of the MWM test. The prevalence rate of rats exhibiting PTSD-like- or mTBI-like responses was calculated from these data. The experimental design is depicted in Fig. 1.

### Blast wave exposure

The experimental tool used an exploding wire technique to generate small-scale cylindrical and spherical blast waves. The exploding wire technique has been demonstrated to simulate the effects of air blast exposure under safe, experimental conditions with repeatability (Ram and Sadot 2012).

#### Exploding wires

To initiate a low-pressure blast wave explosion a current created by a high voltage power supply (4.2 kV) was generated from a capacitor that was delivered to a thin (0.8 mm diameter, 70 mm in length) knotted copper wire. The discharge current was about 500 kA. When the short, high-current pulse passed through the thin conducting wire, it was rapidly heated, expanded and then evaporated. The rapid expansion generated a strong blast wave, whose strength was controlled by the charging voltage. This method of explosion produced a cylindrical blast wave that simulated a blast wave profile similar to that seen from an explosive device common to the battlefield. In an actual explosion the blast wave causes an acute, short-duration elevation in pressure followed by a negative phase. The exploding wire system has been shown to be capable of simulating this overpressure and negative pressure blast wave profile (Ram and Sadot 2012; Zuckerman et al. 2016).

#### Procedure

Each rat was restrained in a custom flexible harness located on a tray, which was then placed in the blast wave generation system at a distance of 265 mm from the wire. Movement was restricted to 3–5 cm during the blast exposure. Pressure values were recorded using a Kistler 211B3 piezoelectric pressure transducer mounted on a perpendicular wall. Non-anesthetized rats were subjected to a single blast wave with the head facing the blast without any body shielding, resulting in a full body exposure to the blast wave. Previous research has reported that this low-pressure blast wave results in a mean peak overpressure of 95 kPa (13.77 psi) (rise time of 0.01 ms) that is sustained for a duration of 0.19 ms and leads to a peak impulse of 10.8 × 10^−3^ kPa s^−1^ (Zuckerman et al. 2016). The overpressure wave is followed with a negative pressure wave that is sustained for more than 0.66 ms with a peak negative pressure of −40 kPa (−5.8 psi). This blast protocol is reported to result in a sound pressure level of 193 dB and a light intensity of approximately 5 Mlux (Zuckerman et al. 2016), which is similar to that experienced during exposure to a M84 stun grenade at a distance of 1.5 m (3.1 Mlux). The peak overpressure deviations between the different experimental trials were between 1 and 3%. Following the blast rats were returned to their home cage. Exposure to this experimental blast wave has recently been validated to elicit distinct behavioral and morphological responses modelling mTBI-like, PTSD-like and comorbid mTBI-PTSD-like behaviors (Zuckerman et al. 2016).

### Neurological severity score (NSS)

To insure that any damage to the central nervous system (CNS) caused by the blast wave did not result in vast neurological deficits, we employed the NSS. The NSS was performed 1-h following the initial blast wave exposure. NSS assesses somatomotor and somatosensory function by evaluating the animals’ activities in motor, sensory, reflexes, beam walking, and beam balancing tasks (Feldman et al. 1996). Specifically, the following were assessed: ability to exit from a circle (3-point scale), gait on a wide surface (3-point scale), gait on a narrow surface (4-point scale), effort to remain on a narrow surface (2-point scale), reflexes (5-point scale), seeking behavior (2-point scale), beam walking (3-point scale), and beam balance (3-point scale). An observer, who was blind to the different treatment groups, tested the animals.

### Behavioral assessments

All rats underwent a number of different behavioral assessments. All behavioral tests were performed in a closed, quiet, light-controlled room in the Faculty of Medicine, Anxiety and Stress Research Unit, Ben-Gurion University between 10:00 and 16:00 h. All results were recorded and analyzed using an EthoVision automated tracking system (Noldus Information Technology, The Netherlands). The behavioral tests included the elevated plus maze and acoustic startle response for anxiety-like/PTSD-like responses and occurred 7-day following initial exposure to the blast wave. The delay in performing these measures from the blast is based upon findings that extreme behavioral changes, which remain constant after 7 days of exposure represent ‘chronic symptoms’ that persist over a prolonged duration (Cohen et al. 2004; Cohen and Zohar 2004).

#### Elevated plus maze (EPM)

Behavioral assessments performed in the EPM have previously been described (Cohen et al. 2003, 2012). The EPM is a plus-shaped platform with two opposing open and two opposing closed arms (open only towards the central platform and surrounded by 14-cm high opaque walls on three sides). Rats were placed on the central platform facing an open arm and allowed to explore the maze for 5 min. Each session was videotaped and subsequently scored by an independent observer. Arm entry was defined as entering an arm with all four paws. Behaviors assessed were: time spent (duration) in open and closed arms and on the central platform; number of open and closed arm entries; and total exploration (entries into all arms). Total exploration was calculated as the number of entries into any arm of the maze to distinguish between impaired exploratory behavior, exploration limited to closed arms (avoidance) and free exploration. “Anxiety index”, an index that integrates the EPM behavioral measures, was calculated as follows:

$${\text{Anxiety index}} = 1 - \frac{{\frac{{ {\text{time spent in the open arms}}}}{\text{total time on the maze}} + \frac{\text{number of entries to the open arms}}{\text{total exploration on the maze}}}}{2}$$

Anxiety index values range from 0 to 1 where an increase in the index expresses increased anxiety-like behavior.

#### Acoustic startle response

Startle response was measured using two ventilated startle chambers (SR-LAB system, San Diego Instruments, San Diego, CA). The SR-LAB calibration unit was used routinely to ensure consistent stabilimeter sensitivity between test chambers and over time. Each Plexiglass cylinder rested on a platform inside a sound-proofed, ventilated chamber. Movement inside the tube was detected by a piezoelectric accelerometer below the frame. Sound levels within each test chamber were measured routinely using a sound level meter to ensure consistent presentation. Each test session started with a 5-min acclimatization period to background white noise of 68 dB, followed by 30 acoustic startle trial stimuli in six blocks (110 dB white noise of 40 ms duration with 30 or 45 s inter-trial interval). Behavioral assessment consisted of mean startle amplitude (averaged over all 30 trials) and percent of startle habituation to repeated presentation of the acoustic pulse. Percent habituation defined as the percent change between the response to the first block of sound stimuli and the last block of sound stimuli was calculated as follows:

$${\text{Percent habituation}} = 100{ \times } \frac{{({\text{average startle amplitude in block}}\; 1) - ({\text{average startle amplictude in block}} \;6)}}{{({\text{average startle amplitude in block}}\; 1)}}$$

#### Morris water maze (MWM)

Spatial learning and memory was assessed by performance in a hippocampal-dependent visuospatial learning task in the MWM according to a test modified from the procedure of Morris (1984). Animals were trained in a pool 1.8 m in diameter and 0.6 m high, filled halfway with water at 24° ± 1 °C. A 10-cm square transparent platform was hidden in a constant position in the pool submerged 1-cm below water level. Within the testing room only distal visual-spatial cues were available to the rats for location of the submerged platform. Rats were given four trials per day to find the hidden platform over four consecutive days (acquisition phase). The escape latency, (i.e., the time required by the rat to find and climb onto the platform), was recorded for up to 120 s. Each rat was allowed to remain on the platform for 30 s, and was then removed to its home cage. If the rat did not find the platform within 120 s, it was manually placed on the platform and returned to its home cage after 30 s. To assess reference memory at the end of learning, a probe trial was given. 24 h after the last acquisition day, the island was removed and the search strategy of the rat was monitored to evaluate whether it used spatial memory to search for the island in the quadrant where it had previously been located. On days 6–7 (days 13–14 from the blast) the platform was placed at the opposite end of the pool, and the rat was retrained in four daily sessions (reversal phase).

### Retrospective classifications and re-analyses by response patterns

#### The cut-off behavioral criteria (CBC) model of PTSD

**Fig. 2:**
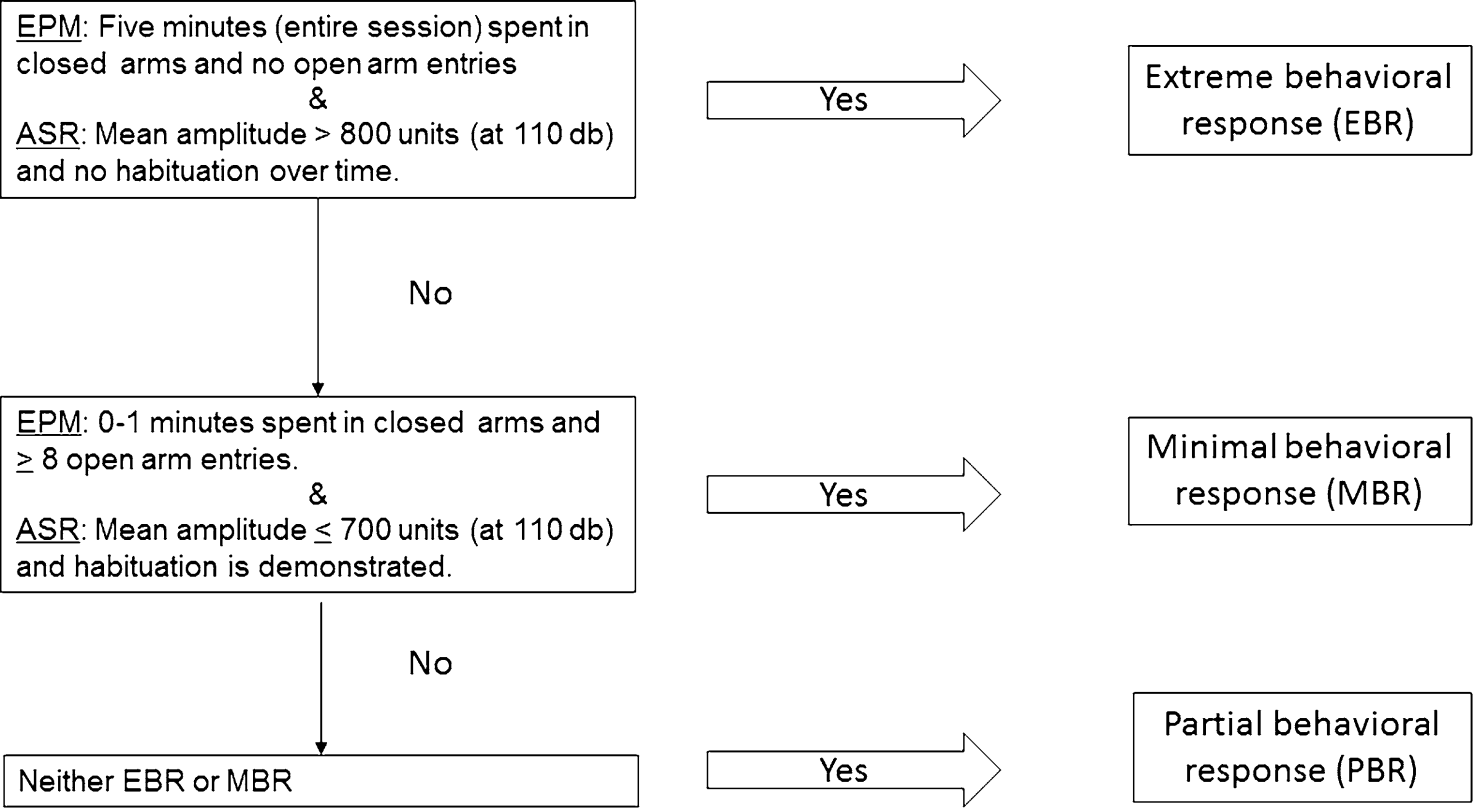
The cut-off behavioral criteria algorithm. Animals were classified into groups according to degree of response to the stressor. *EPM* elevated plus maze, *ASR* acute startle response, *EBR* extreme behavioral response, *MBR* minimal behavioral response, *PBR* partial behavioral response

To model DSM (Diagnostic and Statistical Manual of Mental Disorders) criteria for PTSD, the “the cut-off behavioral criteria” (CBC) model of PTSD-like phenotype was employed (Cohen et al. 2003, 2004; Cohen and Zohar 2004). This model is based on the understanding that a clinical diagnosis of PTSD is made only if an individual exhibits a certain number of symptoms of sufficient severity from well-defined symptom-clusters over a specific period of time. Classifying the degree of how individual behavior is affected by a stressor is based on the premise that an extreme behavioral response to a priming trigger is inadequate and maladaptive, and represents a pathological response (Cohen et al. 2003; Cohen and Zohar 2004). Animals were classified according to their behavioral response pattern on both the EPM and ASR, using the CBC, as exhibiting either an “extreme behavioral response” (EBR) or a “minimal behavioral response” (MBR). Behavioral performances that fulfilled neither set of criteria were labeled as exhibiting a “partial behavioral response” (PBR). This procedure is detailed in Fig. 2.

#### Cognitive performance criteria model of mTBI

Memory loss or impairment is one criterion for human mTBI. As such, we used the animals’ performance in the MWM to evaluate learning and memory (Morris 1984). Examination of escape latencies and time spent in different areas of the pool were used to assess learning and memory. In general, a large degree of variability is seen in response patterns. This is consistent with investigations demonstrated that only a proportion of a population exposed to a blast wave develops symptoms fulfilling mTBI criteria (Doppenberg et al. 2004; Hoge and Castro 2011; Jaffee et al. 2009). Diagnostic criteria that have been previously validated and proven to be reliable were used determine whether an animal exhibited mTBI behavior. Detailed discussion on the cognitive criteria used has been recently reported by Zuckerman et al. (2016). Briefly, the experimental decay parameters, *Y* = *Y*_0_ + *A*/*t*, were calculated from the escape latency determined during the MWM for both acquisition and reversal phases.

$${\text{Escape latency}}\;({\text{EL}}) = {\text{EL}}0 + {\text{EL}}\Delta {\text{e}}( - t / T)$$

**Fig. 3:**
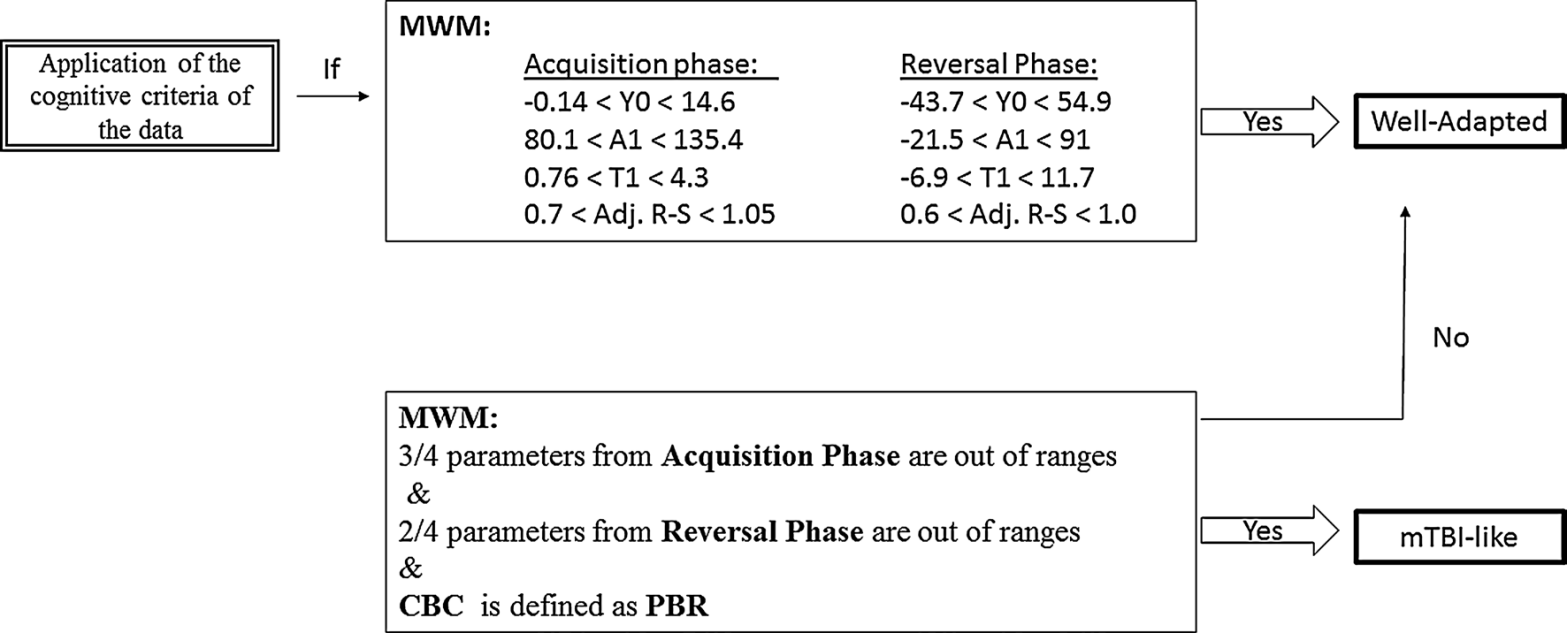
Cognitive criteria algorithm. Animals were classified into groups according to degree of cognitive performance in the Morris water maze

EL refers to the time in seconds it took the rat to reach the platform as a function of input number (number of trials); *Y*_0_ refers to the asymptotic time it took the rat to find the platform for large input values (*t*≫). A refers to the difference (∆) between the time it took the rat to find the platform on the initial input and the asymptotic time (*Y*_0_). *T* refers to the decay constant, a measure of the rat’s rate of learning. The slope at the initial input (*A*/*t*) is the initial learning speed, and the adjusted *R* ^2^ refers to the goodness of fit. Where EL0 = the asymptotic value of EL as *t* = end point or EL final; ELΔ = the difference between EL peak and EL0; *t* = number of inputs to the maze. According to the means of the exponential decay parameters (EL0, ELΔ, *T* and adjusted *R* ^2^), confidence limit for “normal performance” was defined for each variable (±2 standard deviations) for acquisition and reversal phases. Subsequently, the memory performance obtained by fitting the escape latency decay curve was used to assess learning and memory performance after blast exposure. Individual animals were classified according to their cognitive and behavioral response pattern, as exhibiting either “mTBI-related” or well-adapted memory performance. The procedure is detailed in Fig. 3.

### Tissue preparation

All animals were euthanized 24 h after the last behavioral tests. Animals were deeply anesthetized via an intraperitoneal injection of a ketamine and xylazine mixture (70, 6 mg kg^−1^, respectively) and perfused transcardially with cold 0.9% physiological saline followed by 4% paraformaldehyde (Sigma-Aldrich) in 0.1 M phosphate buffer (pH 7.4). Brains were quickly removed, postfixed in the same fixative for 12 h at 4 °C, and were cryoprotected overnight in 30% sucrose in 0.1 M phosphate buffer at 4 °C. Brains were frozen on dry ice and stored at −80 °C. Serial coronal sections (10 µm) at the level of dorsal hippocampus were collected for each animal, using a cryostat (Leica CM 1850) and mounted on coated slides.

### Immunofluorescence

Sliced sections were air dried and incubated in frozen methanol (2 min) and in 4% paraformalaldehyde (4 min). After three washes in phosphate buffer saline (PBS) containing Tween 20 (PBS/T) (Sigma-Aldrich), the sections were incubated for 60 min in a blocking solution (normal goat serum, in PBS) and then overnight at 4 °C with the primary antibodies against neuropeptide Y (NPY) (mouse monoclonal anti-NPY antiserum (1:500), product code: sc-133080, Santa Cruz Biotechnology, Inc. Heidelberg Germany), brain-derived neurotrophic factor (BDNF) (rabbit polyclonal anti-BDNF antiserum (1:300), product code: sc-ANT-010, Alomone Labs, Jerusalem Israel), glial fibrillary acidic protein (GFAP) (mouse polyclonal anti-GFAP antiserum (1:200), product code: G3893, Sigma-Saint Louis, MO, USA) and tau protein (rabbit polyclonal anti-tau antiserum (1:1000), product code: Ab-64193, Abcam Israel). After three washes in PBS/T, sections were incubated for 2 h in DyLight-488 labeled goat anti-rabbit IgG (BNDF: 1/1250; tau: 1/1750) or in Dylight-594 goat anti-mouse IgG (NPY: 1/500; GFAP: 1/500; KPL, MD, USA) in PBS containing 2% normal goat or horse serum.

### Quantification

A computer-assisted image analysis system (Leica Application Suite V3.6, Leica, Germany) was used for quantitative analysis of the immunostaining and 50× objective lens were employed to assess the number of BDNF-IR, NPY-IR, GFAP-IR, and tau-IR protein cells in the hippocampus, divided into three (counted separately) areas: CA1 subfield, CA3 subfield and dentate gyrus (DG). The regions of interest were outlined and computer-aided estimation was used to calculate the number of BDNF-IR, NPY-IR, GFAP-IR, and tau-IR protein cells in the pyramidal layer of CA1 and CA3, and in the granular layer of the DG. Seven representative sections of the hippocampus were chosen (between Bregma −2.30 and Bregma −3.60) from each animal, from each group. The sections were analyzed by two observers blinded to the treatment protocol. Standard technique was used to estimate the number of NPY, BDNF, GFAP, and tau protein cell profiles per unit area for each investigated hippocampal structure.

### Measurement of brain carnosine and histidine content

Carnosine and histidine content in brain homogenates were determined by high performance liquid chromatographic/tandem mass spectrometric (HPLC/MS/MS) analysis according to previously published methods (Casetta et al. 2000). Brains were partially thawed on ice and five brain regions were sampled: cerebral cortex, hypothalamus, hippocampus, amygdala, and thalamus. Each sample was weighed and transferred into individual vials for further homogenization. Samples were analyzed using HPLC–MS/MS with positive electrospray ionization for histidine and carnosine. Quantification of histidine and carnosine was performed using a deuterium labeled internal standard (IS), l-alanine-d4 and dipeptide alanyl-glutamine. The proteins in the brain tissue samples were removed by the protein precipitation with methanol. After protein precipitation the brain tissue samples were derivatized with 3 M hydrogen chloride 1-butanol solution. After addition of internal standards and centrifugation the samples were injected onto the HPLC–MS/MS system. All samples were analyzed separating them first by reversed phase gradient LC and subsequently detecting them using electrospray ionization and multiple reaction monitoring (MRM).

### Statistical analyses

To compare the effect of β-alanine ingestion on the prevalence of animals exhibiting mTBI-like, PTSD-like and the comorbid mTBI-PTSD-like characteristics a *χ* ^2^ analysis was used. Comparisons of brain carnosine and histidine content between BA and PL were performed using an unpaired Student’s *t* test. A one-way analysis of variance (ANOVA) was employed to compare immunofluorescence differences of BDNF, NPY, GFAP and tau protein between BA, PL and CTL. In the event of a significant *F* ratio, LSD post hoc analysis was used for pair-wise comparisons. Pearson’s product-moment correlation was used to determine selected bivariate correlations. Data were analyzed using SPSS v22 software (SPSS Inc., Chicago, IL). All data are reported as mean ± SD. An alpha level of *p* < 0.05 was used to determine statistical significance.

## Results

### NSS

No significant differences between BA and PL were noted in reflex responses, motor coordination, motor strength, or sensory function (data not shown). These findings indicate that differences between behavioral and cognitive tasks were not related to abnormal motor function required of the animals to complete the behavioral tasks.

### Prevalence rates of mTBI, PTSD and mTBI + PTSD according to the behavioral and cognitive performance criteria

**Fig. 4:**
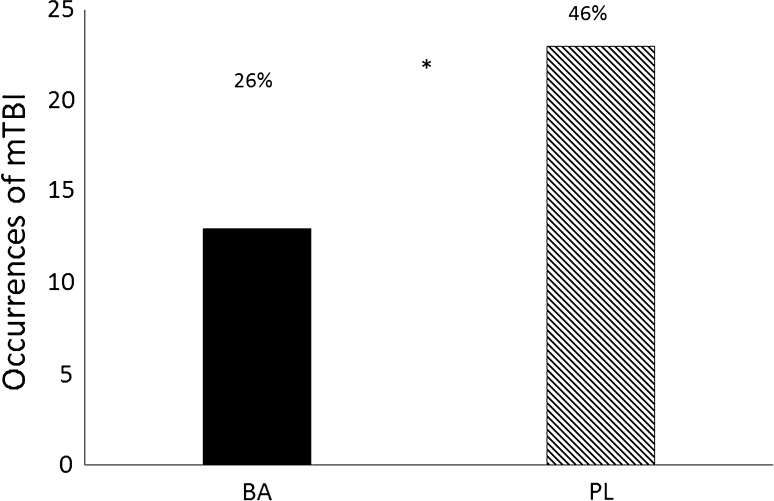
Prevalence rate of animals exhibiting mTBI-like behavior. *BA* β-alanine, *PL* placebo; *significant difference between the groups

Significant differences were found between groups in the occurrence of animals fulfilling the criteria for mTBI (*χ* ^2^ = 4.05, *p* = 0.044) (see Fig. 4). The prevalence of animals demonstrating mTBI was significantly lower in BA than in PL treated rats [26.5% (13/49) and 46% (23/50), respectively]. No significant differences were noted between the groups in the prevalence of animals displaying PTSD-like patterns of behavior (*χ* ^2^ = 0.007, *p* = 0.93). The prevalence of PTSD was similar between BA and PL treated rats [10.2% (5/49) and 12.0% (6/50), respectively]. In addition, the prevalence of animals demonstrating the comorbid pattern of mTBI + PTSD was not significantly different (*χ* ^2^ = 1.31, *p* = 0.25) between BA (4%, 2/49) and PL (10%, 5/50). A trend though was noted (*χ* ^2^ = 3.07, *p* = 0.08) in the prevalence of well-adapted animals between the groups. Animals in BA exposed to the low-pressure blast wave tended to be more well adapted (67%, 33/49) than animals in PL (50%, 25/50).

### BDNF expression at day 16 following blast exposure

**Fig. 5:**
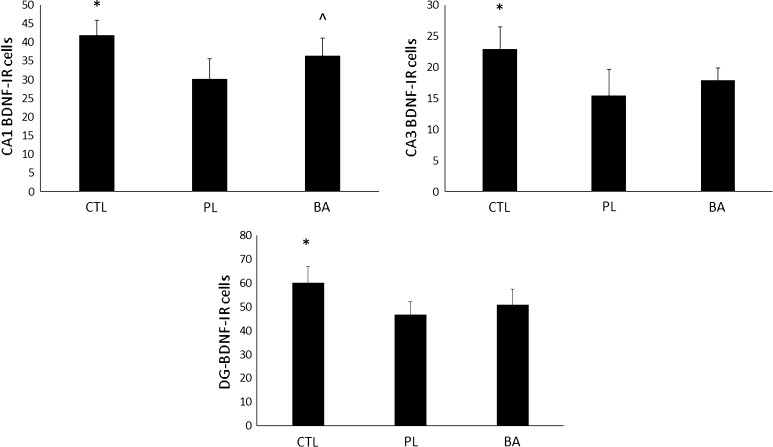
BDNF expression at day 16 post-blast exposure. *Significantly different than PL and BA; ^significantly different than PL. *CTL* control group consisting of animals that were fed a normal diet and not exposed to the blast, *PL* animals that were fed a normal diet and were exposed to the blast, *BA* animals that were supplemented with β-alanine and exposed to the blast. All data reported as mean ± SD

Comparisons between BA, PL and CTL for BDNF expression in the CA1, CA3 and DG subregions can be observed in Fig. 5a–c, respectively. Significant differences were noted in the CA1 [*F* (2, 25) = 12.9, *p* < 0.001], CA3 [*F* (2, 25) = 11.1, *p* < 0.001] and DG [*F* (2, 25) = 10.5, *p* < 0.001] subregions. BDNF expression in the CA1 subregion of animals not exposed to the blast and fed a normal diet (CTL) were significantly higher than both PL (*p* < 0.001) and BA (*p* = 0.025). BDNF expression in animals that were exposed to the blast and supplemented with β-alanine were significantly higher than PL (*p* = 0.009). In addition, BDNF expression in the CA3 subregion in CTL was significantly greater than both BA (*p* = 0.005) and PL (*p* < 0.001). No difference was noted in BDNF expression in CA3 between BA and PL (*p* = 0.110). In the DG subregion, BDNF expression in CTL was significantly greater than both BA (*p* = 0.005) and PL (*p* < 0.001), while no differences were noted in BDNF expression in the DG subregion between BA and PL (*p* = 0.136).

### NPY expression at day 16 following blast exposure

**Fig. 6:**
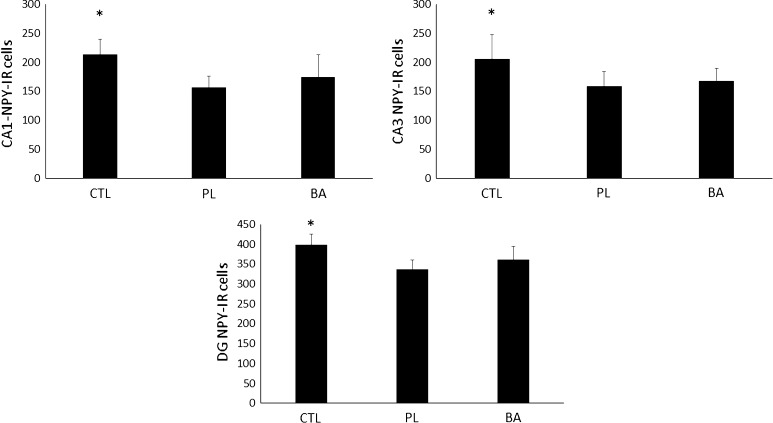
NPY expression at day 16 post-blast exposure. *Significantly different than PL and BA. *CTL* control group consisting of animals that were fed a normal diet and not exposed to the blast, *PL* animals that were fed a normal diet and were exposed to the blast, *BA* animals that were supplemented with β-alanine and exposed to the blast. All data reported as mean ± SD

Comparisons between BA, PL and CTL for NPY expression in the CA1, CA3 and DG subregions are depicted in Fig. 6a–c, respectively. Significant differences were noted in the CA1 [*F* (2, 25) = 8.3, *p* = 0.002], CA3 [*F* (2, 25) = 5.9, *p* = 0.008] and DG [*F* (2, 25) = 10.2, *p* = 0.001] subregions. NPY expression in the CA1 subregion of CTL were significantly higher than BA (*p* = 0.011) and PL (*p* < 0.001). No significant differences though were noted between BA and PL (*p* = 0.196). NPY expression in the CA3 subregion for CTL was significantly greater than both BA (*p* = 0.032) and PL (*p* = 0.003), and again no differences were noted between BA and PL (*p* = 0.480). In the DG subregion, NPY expression in CTL was significantly greater than both BA (*p* = 0.011) and PL (*p* < 0.001), while NPY expression in BA trended (*p* = 0.074) towards a higher response compared to PL.

### GFAP expression at day 16 following blast exposure

**Fig. 7:**
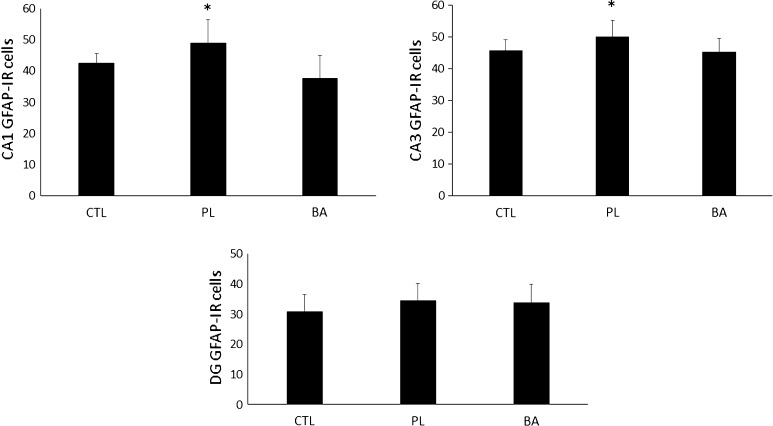
GFAP expression at day 16 post-blast exposure. *Significantly different than CTL and BA. *CTL* control group consisting of animals that were fed a normal diet and not exposed to the blast, *PL* animals that were fed a normal diet and were exposed to the blast, *BA* animals that were supplemented with β-alanine and exposed to the blast. All data reported as mean ± SD

Comparisons between BA, PL and CTL for GFAP expression in the CA1, CA3 and DG subregions are depicted in Fig. 7a–c, respectively. Significant differences were noted in the CA1 [*F* (2, 25) = 7.6, *p* = 0.003] and CA3 [*F* (2, 25) = 3.7, *p* = 0.040] subregions, but no difference was noted in the DG [*F* (2, 25) = 0.99, *p* = 0.387] subregion. GFAP expression in the CA1 subregion of PL was significantly greater than BA (*p* = 0.001) and CTL (*p* < 0.047). No differences were noted between CTL and BA (*p* = 0.126). GFAP expression in the CA3 subregion for PL was significantly greater than both BA (*p* = 0.021) and CTL (*p* = 0.040), but no differences were noted between BA and CTL (*p* = 0.876).

### Tau protein expression at day 16 following blast exposure

**Fig. 8:**
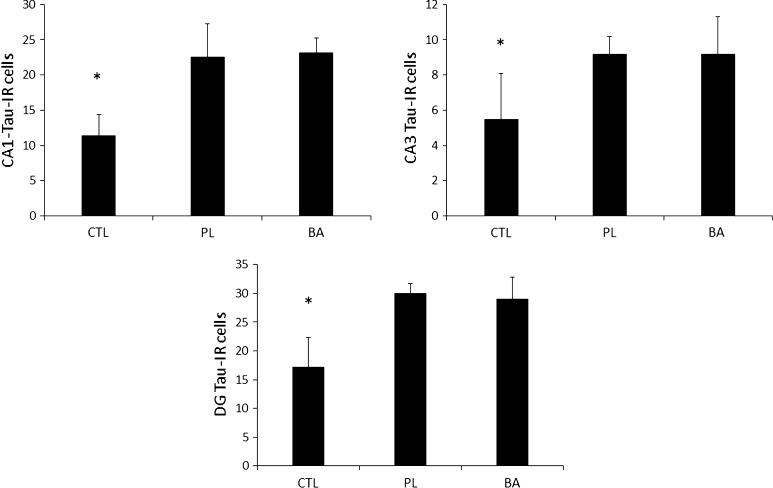
Tau protein expression at day 16 post-blast exposure. *Significantly different than PL and BA. *CTL* control group consisting of animals that were fed a normal diet and not exposed to the blast, *PL* animals that were fed a normal diet and were exposed to the blast, *BA* animals that were supplemented with β-alanine and exposed to the blast. All data reported as mean ± SD

Comparisons between BA, PL and CTL for tau protein expression in the CA1, CA3 and DG subregions are depicted in Fig. 8a–c, respectively. Significant differences were noted in the CA1 [*F* (2, 27) = 37.2, *p* < 0.001], CA3 [*F* (2, 27) = 11.0, *p* < 0.001] and DG [*F* (2, 27) = 36.5, *p* < 0.001] subregions. Tau protein expression in the CA1 subregion of CTL were significantly lower than both BA (*p* < 0.001) and PL (*p* < 0.001). No significant differences though were noted between BA and PL (*p* = 0.700). Tau protein expression in the CA3 subregion for CTL was also significantly lower than both BA (*p* < 0.001) and PL (*p* < 0.001), and again no differences were noted between BA and PL (*p* = 1.000). In the DG subregion, tau protein expression in CTL was significantly lower than both BA (*p* < 0.001) and PL (*p* < 0.001), with no differences observed (*p* = 0.557) between BA and PL.

### Brain carnosine and histidine content at day 16 following blast exposure

**Fig. 9:**
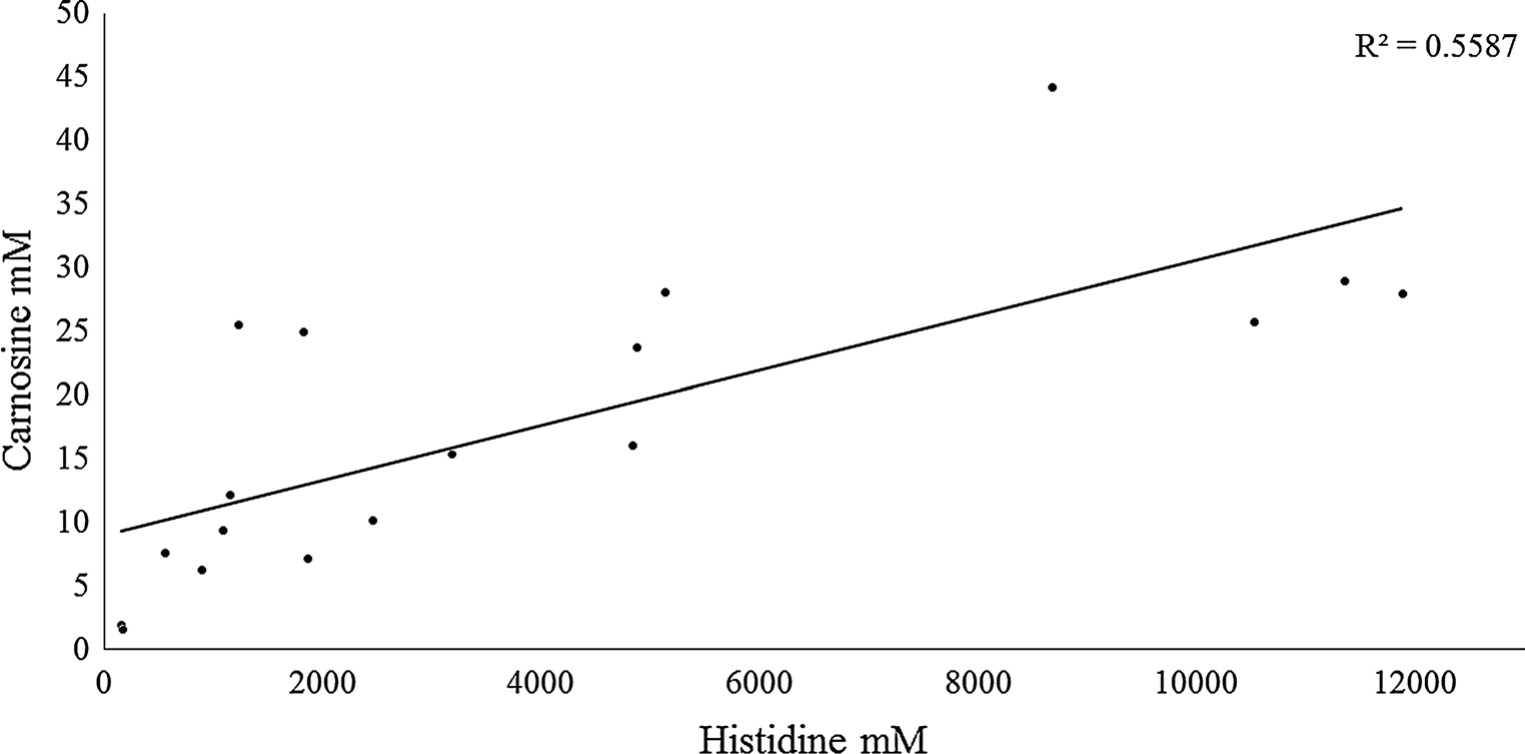
Relationship between changes in carnosine and histidine (mM) content in the hippocampus

Differences in carnosine and histidine content in various areas of the brain can be observed in Table 1. Across the five brain regions analyzed the carnosine content in animals that consumed β-alanine was on average 79% higher than in those animals that consumed the vehicle. Carnosine content in the cerebral cortex was significantly higher (*p* = 0.048) for BA compared to PL. Trends towards a difference were also seen in the hippocampus (*p* = 0.058) and amygdala (*p* = 0.061). Histidine content across the five regions of the brain that were analyzed was 86% higher for BA than P. Trends towards a higher histidine content were noted in the hippocampus (*p* = 0.053), cerebral cortex (*p* = 0.070) and thalamus (*p* = 0.108) for BA compared to PL. Carnosine content was significantly correlated (*r* = 0.75, *p* < 0.001) to histidine content in the hippocampus (see Fig. 9). Positive correlations were also noted between carnosine and histidine in the cortex (*r* = 0.48, *p* = 0.037), hypothalamus (*r* = 0.48, *p* = 0.036) and thalamus (*r* = 0.43, *p* = 0.070).

**Table 1:** Brain carnosine and histidine concentrations (mM)

	Hippocampus	Cortex	Hypothalamus	Amygdala	Thalamus
Carnosine					
BA	22.4 ± 11.3	364 ± 178	22.7 ± 26.2	23.2 ± 18.4	50.9 ± 33.9
PL	12.7 ± 8.4	213 ± 110	9.5 ± 6.7	10.4 ± 4.4	57.5 ± 63.1
*p* value	0.058	0.048*	0.166	0.061	0.795
Histidine					
BA	5632 ± 4995	5669 ± 4471	1862 ± 1794	2619 ± 1467	26,025 ± 21,754
PL	2768 ± 1771*	2128 ± 1537	1144 ± 794	3029 ± 1582	12,141 ± 12,523
*p* value	0.053	0.070	0.266	0.576	0.108

All data reported as mean ± SD

* Significant difference between groups

## Discussion

The results of this study indicated that 30-day of β-alanine ingestion in rats was effective in reducing the incidence of mTBI-like phenotype following exposure to a low-pressure blast wave. Animals supplemented with β-alanine and exposed to the blast wave also appeared to have a reduced inflammatory response and a higher BDNF expression in specific regions of the hippocampus compared to rats that were exposed, but fed a normal diet. Exposure to the blast wave resulted in significant elevations in tau protein expression and significant decreases in NPY expression, regardless of β-alanine ingestion.

The low-pressure blast wave used in this study has been previously demonstrated to be an effective model to elicit distinct behavioral and morphological changes that simulate mTBI-like, PTSD-like, and comorbid mTBI-PTSD-like responses (Zuckerman et al. 2016). The blast wave that was generated from the detonation of the thin knotted copper wire resulted in a spherical blast wave, which included a typical overpressure peak and underpressure wave common to an improvised or standard explosive device (Ram and Sadot 2012). This blast profile has been demonstrated to have a typical heterogeneous response in behavioral and memory outcomes in animals exposed to the single blast (Ram and Sadot 2012). In this study, the cognitive/behavioral response patterns observed in animals exposed to the low-pressure blast wave and fed a normal diet, resulted in 46% (23/50) of these animals exhibiting mTBI-like responses. This was slightly greater than the incident rate previously reported [34.6% (19/55)] using the same blast model (Zuckerman et al. 2016). Supplementation with β-alanine appeared to increase the resiliency to mTBI-like response patterns. Rats’ who were fed β-alanine for 30 days had a significantly lower incident rate [26.5% (13/49)] of mTBI than the animals fed a normal diet. Considering that the symptoms associated with mTBI and PTSD are quite similar (Bryant et al. 2010; Elder et al. 2012; Ojo et al. 2014; Pape et al. 2013), it was hypothesized that attenuation of the occurrence of mTBI-like symptoms would also be associated with similar resiliency in PTSD-like effects.

Previously, we have demonstrated that 30-day of β-alanine supplementation can increase resiliency to an animal model of PTSD (Hoffman et al. 2015b). The protective effects associated with elevations in brain carnosine were suggested to be related to a protection of BDNF expression in the hippocampus. Although a similar response regarding maintaining BDNF expression was noted in this study, no differences in PTSD-like behavior was observed between the groups exposed to the low-pressure blast wave. These differences may be related to the different models used between the studies. The previous study used a predator scent stress (PSS) which is a validated animal model of PTSD (Cohen and Zohar 2004). Although the low-pressure blast wave model has been demonstrated to result in PTSD-like symptoms (Zuckerman et al. 2016), it was primarily developed to cause cognitive and behavioral deficits simulating mTBI-like symptoms. Incident rates of extreme behavioral responses using the PSS have been reported to vary between 22 and 55% (Cohen et al. 2010; Cohen and Zohar 2004). In contrast, the low-pressure blast wave has been previously shown to elicit an extreme behavioral response in 5% of the rats exposed to the blast (Zuckerman et al. 2016). Although the incident rate of PTSD-like response appeared to increase twofold in this study, it was still below that observed in other studies using the PSS model (Cohen et al. 2010; Cohen and Zohar 2004).

A significant elevation in brain carnosine content was noted in the cortex of animals that supplemented with β-alanine. In addition, strong trends towards an elevation were observed in both the hippocampus and amygdala. We have previously demonstrated significant elevations in all brain regions following a similar dosing protocol in rats (Hoffman et al. 2015b). Whether exposure to the low-pressure blast wave had any influence on brain carnosine content cannot be discerned from the present results. However, the significant elevation observed in histidine content in the hippocampus, and the trend towards an increase in the cortex and thalamus does present an interesting outcome. Carnosinase is an enzyme that degrades carnosine into β-alanine and histidine (Bellia et al. 2014). It is found in very low concentrations in skeletal muscle, but in much larger concentrations in the brain (Kunze et al. 1986; Teufel et al. 2003). It is possible that elevated carnosine content in the hippocampus, resulting from β-alanine supplementation, was metabolized leading to increased levels of both histidine and β-alanine. This appears to be the first study to demonstrate a linear relationship between elevations in hippocampal carnosine and histidine levels. Although the β-alanine content in the different brain regions was not measured, increases in the histidine pool in the hippocampus and other areas suggests that increases in β-alanine content may have occurred as well. It is possible that elevations in both carnosine and β-alanine may have influenced the results observed in this study. Recently, evidence has been presented indicating that β-alanine can act as a neurotransmitter in the brain (Tiedje et al. 2010; Chesnoy-Marchais 2016). Specifically, it has been reported to interact with glial γ-aminobutyric acid (GABA) uptake transporter mechanisms (Tiedje et al. 2010). A recent study demonstrated that β-alanine can reverse the blocking action of GABA receptors suggesting that β-alanine itself may have a neuroprotective role during damaging situations (Chesnoy-Marchais 2016). Although speculative, it is possible that the increased resiliency of mTBI-like behavior may have been the result of the combined effects of both increased carnosine and possibly β-alanine content in the hippocampus.

The role of BDNF as both a neuroprotector and neurotrophin make it uniquely positioned to modulate the acute response to neurotrauma. Previous studies have reported significant decreases in the expression of BDNF in the hippocampus in animals exhibiting PTSD-like behavior (Kozlovsky et al. 2007; Zohar et al. 2011), and a preservation of BDNF expression in animals that appeared to be more resilient to similar stressors (Cohen et al. 2012; Hoffman et al. 2015a, b). In this study, expression of BDNF in the CA1 subregion of the hippocampus was significantly higher in BA compared to PL. However, exposure to the blast still resulted in significant decreases in BDNF expression in all animals, regardless of β-alanine ingestion. Although data is limited in animal models, human studies have reported that specific BDNF genotypes are related to a greater occurrence of mTBI in servicemen who returned from deployment (Dretsch et al. 2016; McAllister et al. 2012). BDNF polymorphism may impact hippocampal dendritic morphology influencing the brains’ susceptibility to traumatic injury (McAllister et al. 2012). Changes in BDNF expression in the CA1, but not in the CA3 or DG subregions, consequent to β-alanine ingestion, is consistent with previous research using a similar duration of β-alanine supplementation (Hoffman et al. 2015b). Expression of BDNF has been implicated in the process of memory consolidation (Ozawa et al. 2014). Improved learning outcomes during the MWM assessment in BA may have been partly related to the greater BDNF expression in the CA1 subregion of these animals compared to PL. Although upregulation in BDNF expression in the CA1 subregion has been associated with greater resiliency to stress (Cohen et al. 2014; Kozlovsky et al. 2007), these studies used an animal model of PTSD. This study provides evidence that even by partially maintaining BDNF expression in the CA1 subregion, an increase in resiliency to a low-pressure blast wave may be observed.

Significant reductions in the expression of NPY was noted in the CA1, CA3 and DG subregions of the hippocampus in rats exposed to the low-pressure blast wave, regardless of supplement ingestion. This response was similar to that reported in rats exhibiting PTSD-like behavior (Cohen et al. 2012; Hoffman et al. 2015a). NPY is one of the most abundant neuropeptides in both the central and peripheral nervous system, and has an important role in both neuroprotection and neurogenesis (Malva et al. 2012). Evidence is becoming clear that NPY is associated with learning and memory. When NPY is administered to animals exposed to a neurodegenerative stress (e.g., PTSD or Alzheimer’s disease), attenuation of anxiety (Cohen et al. 2012) and memory loss (dos Santos et al. 2013) has been reported. In addition, decreases in NPY expression during aging is also associated with learning and memory impairments (Hattiangady et al. 2005). This appears to be the first study to examine the effect of a low-pressure blast wave on changes in NPY expression in the hippocampus. Rats supplemented with β-alanine had a significantly greater resiliency to the blast wave despite significant reductions in NPY expression, suggesting that the mechanisms involved in learning and memory are quite complicated and are not dependent upon a single neurotrophin or neuropeptide. Interestingly, NPY has been suggested to be a mediator of BDNF-induced synaptic plasticity and cognitive function including spatial memory (Koponen et al. 2004). Considering that BDNF expression was partially maintained in the CA1 subregion in β-alanine supplemented rats, it is likely that the greater expression of BDNF in this subregion, independent of NPY expression, in the animals ingesting β-alanine contributed to the increased resiliency to the low-pressure blast wave. The importance of seeing this in the CA1 subregion may be related to its role in mediating temporal processing of information including spatial memory (Kesner et al. 2004).

Supplementation with β-alanine was associated with attenuating GFAP expression in the CA1 and CA3 subregions of the hippocampus. Elevations in GFAP are often reported following both traumatic and mild traumatic brain injury (Diaz-Arrastia et al. 2014; Hylin et al. 2013; Kochanek et al. 2006; Perez-Polo et al. 2015; Yang et al. 2013). An insult to brain tissue, such as that which may be experienced from a blast wave, results in both molecular and morphologic changes in microglia and astrocytes that leads to a secretion of inflammatory cytokines (Sajja et al. 2016). These changes occur immediately following injury, but are also sustained as a result of elevations in reactive oxygen species, which exacerbates astrocyte activation increasing GFAP expression (Sajja et al. 2014, 2016). It is this response that β-alanine ingestion may provide a degree of protection. Previous research has suggested that carnosine elevation may serve as a neural protectant through its action as an antioxidant (Kohen et al. 1988). We have recently reported that elevations in carnosine within the hippocampus were associated with increasing resiliency to PTSD in an animal model of PTSD (Hoffman et al. 2015b). Although inflammatory markers were not examined in the former study, this present investigation does provide evidence that elevations in carnosine content in the hippocampus may be associated with attenuating the inflammatory response to a low-pressure blast wave.

Elevations in the expression of tau protein in the CA1, CA3 and DG subregions are consistent with reports from other investigations demonstrating blast-induced brain injury (Du et al. 2016; Goldstein et al. 2012). Increases in tau protein expression occurred in both BA and PL, indicating that the protective effects associated with carnosine’s possible role as an antioxidant did not attenuate tau protein expression. This is in contrast to a recent investigation by Du et al. (2016) who reported that a combination of the antioxidants *n*-acetylcysteine (300 mg kg^−1^) and 2,4-disulfonyl α-phenyl tertiary butyl nitrone (300 mg kg^−1^) intraperitoneally injected into animals following blast exposure, significantly reduced the expression of tau protein in the DG. However, these investigators did not report on any behavioral or memory changes. The resiliency seen for mTBI in animals supplemented with β-alanine was noted despite elevations in tau protein expression, suggests that elevations in tau protein from a single blast may not lead directly to impaired spatial memory. Recent evidence indicates that a greater degree of neuropathology is observed from multiple blast exposures rather than a single blast exposure (Meabon et al. 2016). Thus, elevations in brain carnosine may provide resiliency to an isolated or single exposure to a low-pressure blast wave, despite elevations observed in tau protein expression. Whether a similar degree of resiliency can be observed following multiple blast exposures is unknown.

There are several limitations with this study. Despite a significantly lower incidence rate of mTBI-like behavior in BA, no changes were noted in NPY and tau protein expressions. Although GFAP expression was attenuated as a result of β-alanine ingestion, further examination of additional inflammatory and oxidative stress markers should be investigated. This study was unable to confirm our previous work demonstrating an increased resiliency of β-alanine supplementation on PTSD-like behavior. However, the model used was different, and the incident rate of animals exhibiting PTSD-like behavior did not provide the statistical power to provide any meaningful comparison. Still, rats supplemented with β-alanine and exposed to the low-pressure blast wave tended to be more well adapted than PL. In conclusion, the results of this study provide initial evidence that 30-day of β-alanine supplementation increases resiliency to mTBI-like responses in animals exposed to a low-pressure blast wave.
